# Supplementary material for: FAT10 is phosphorylated by IKKβ to inhibit the antiviral type-I interferon response
Source: Life Sci Alliance. 2023 Nov 8;7(1):e202101282. doi: 10.26508/lsa.202101282 (PMC10631552; doi:10.26508/lsa.202101282)

Raw images of the manuscript-

FAT10 is phosphorylated by IKKβ to inhibit the antiviral type-I interferon response

Figure 1A

Fig 1A

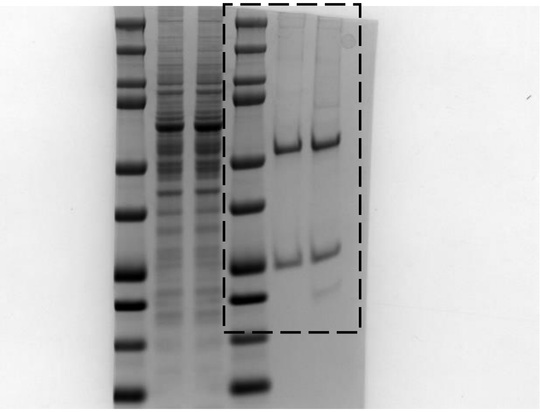

Coomassie

IB: FAT10

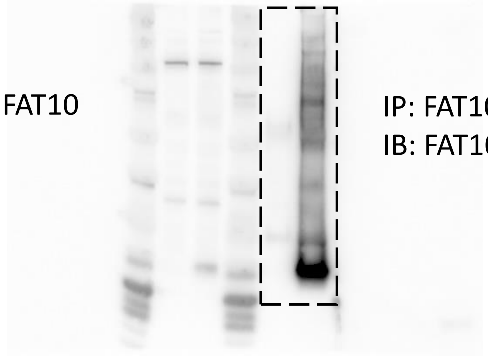

IP: FAT10
IB: FAT10

(longer exposure time)

IB: FAT10

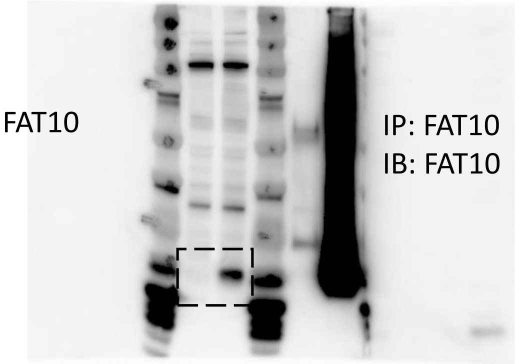

IP: FAT10
IB: FAT10

IB: β-actin

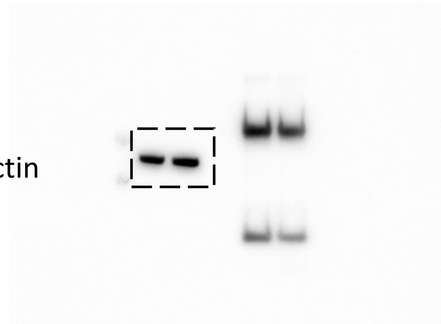

Figure 1C

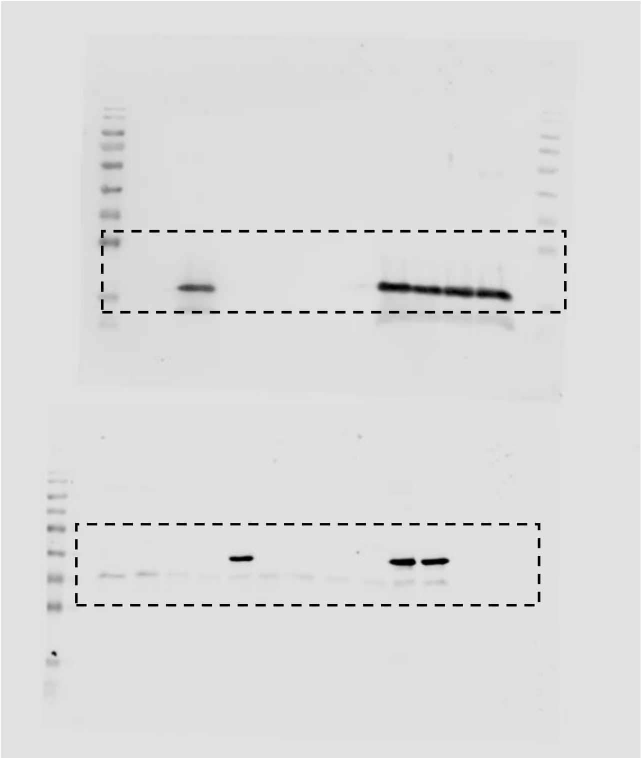

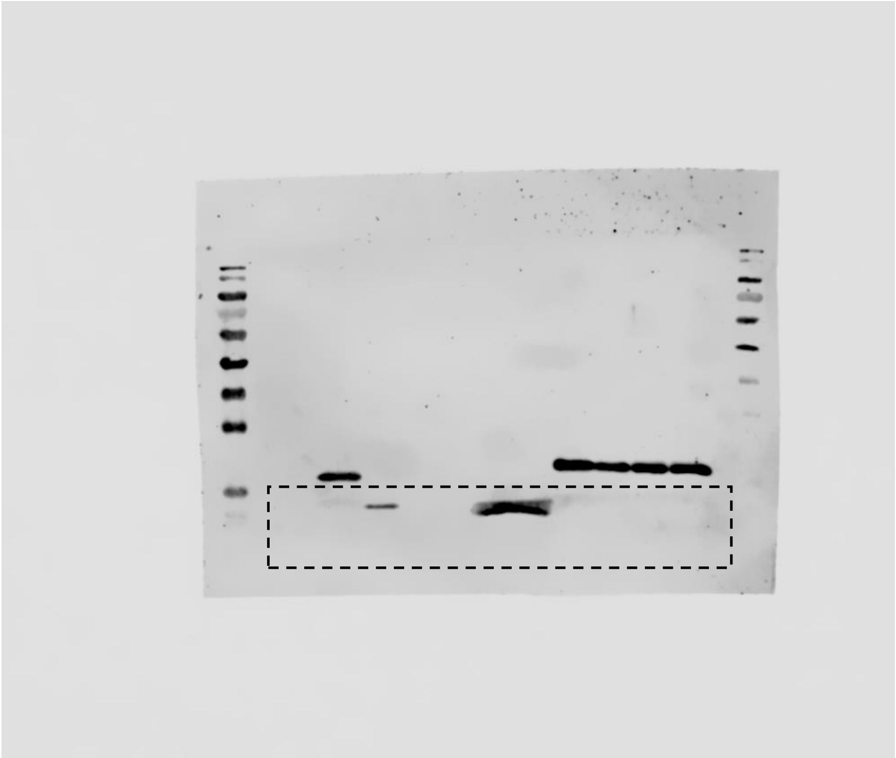

Figure 2A

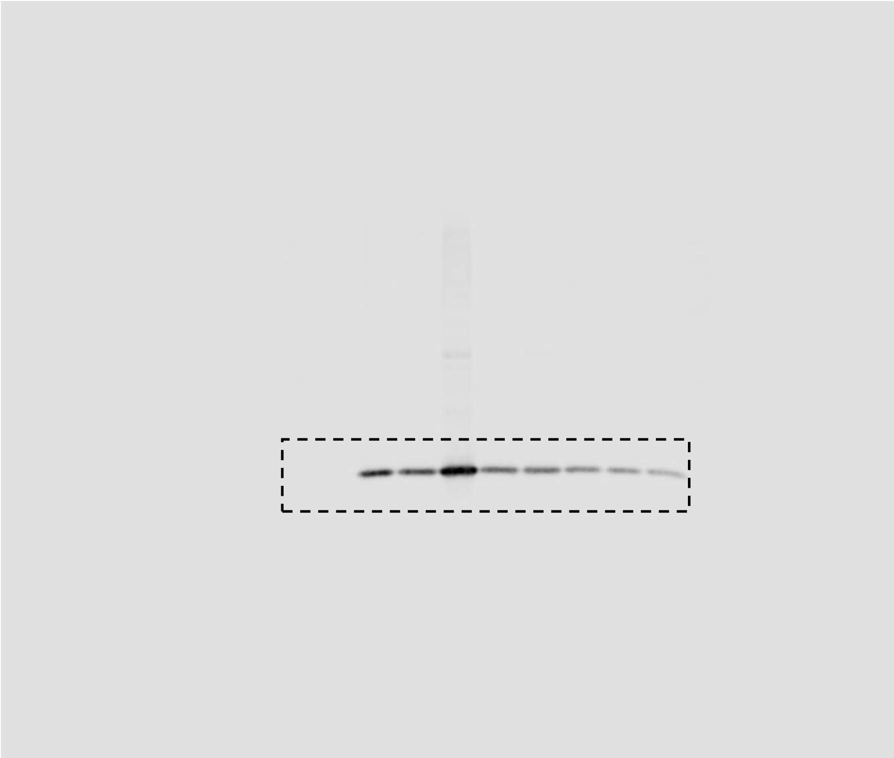

IB: FLAG,
Load

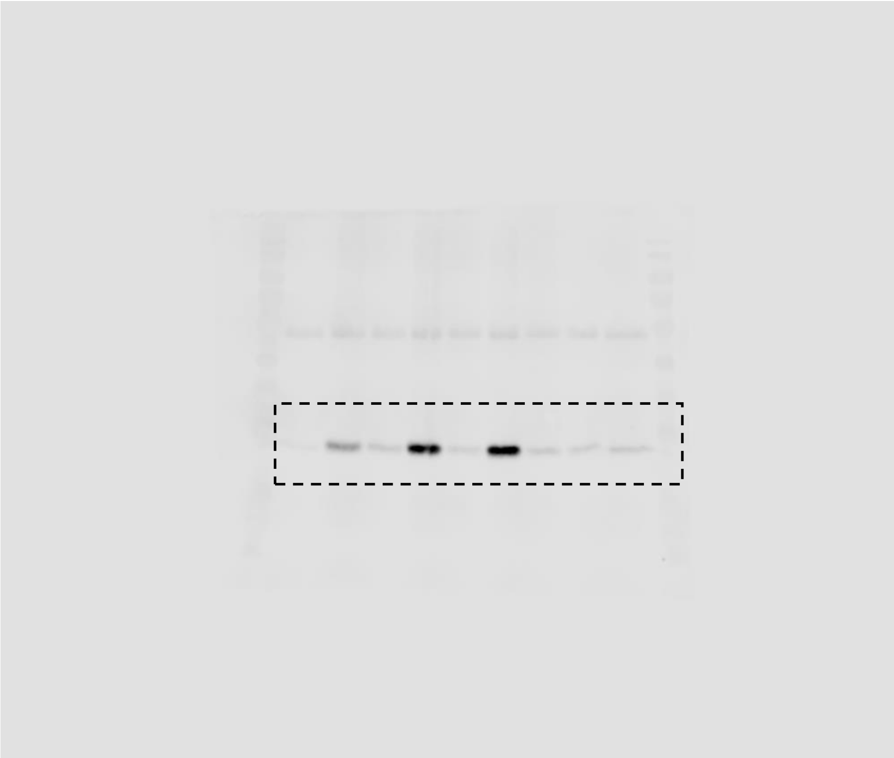

IP: p-Ser
IB: FLAG

Figure 2A

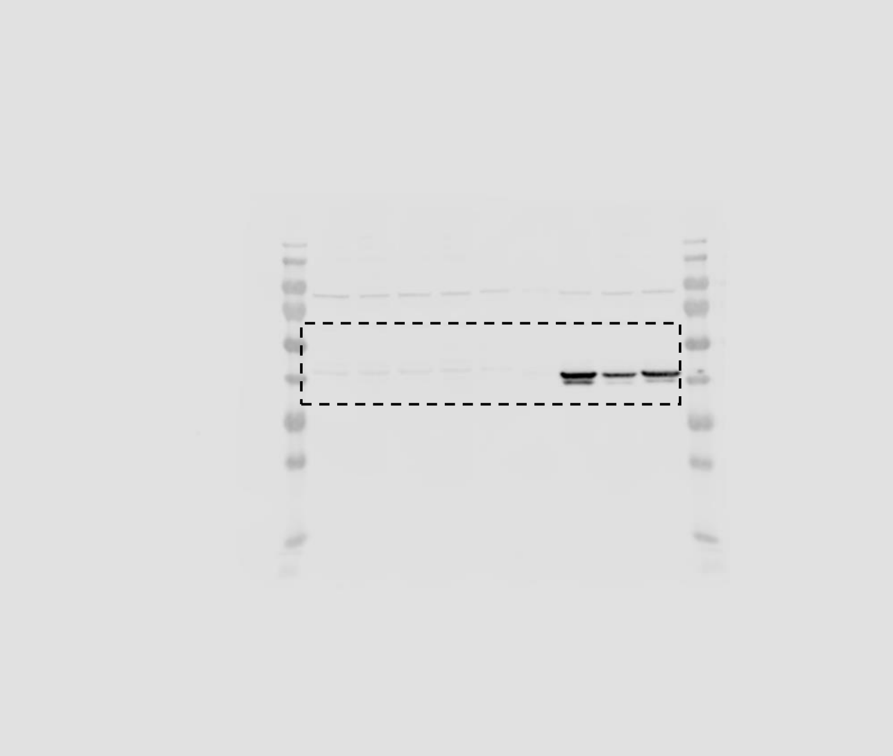

IB: HA,
Load

Figure 2B

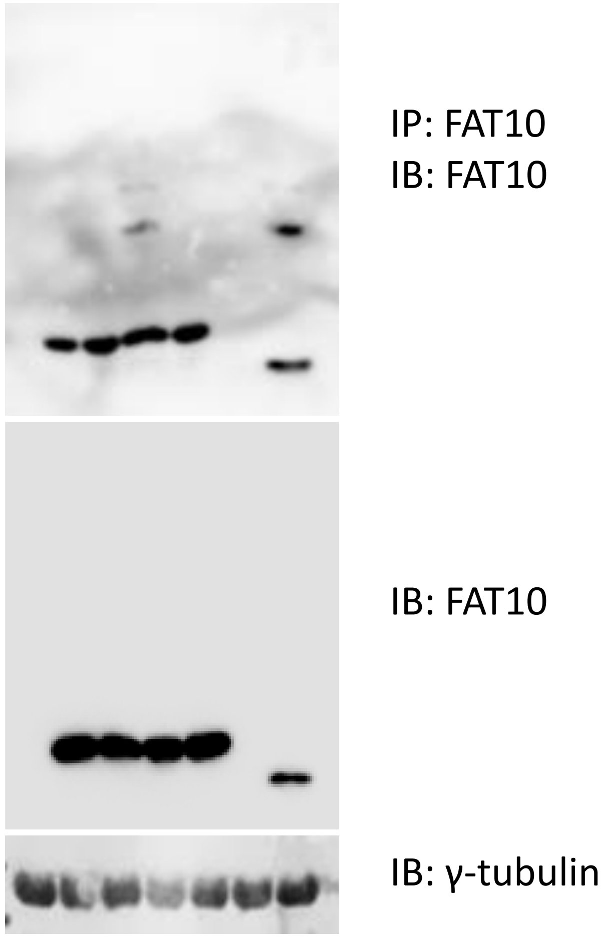

Figure 2C

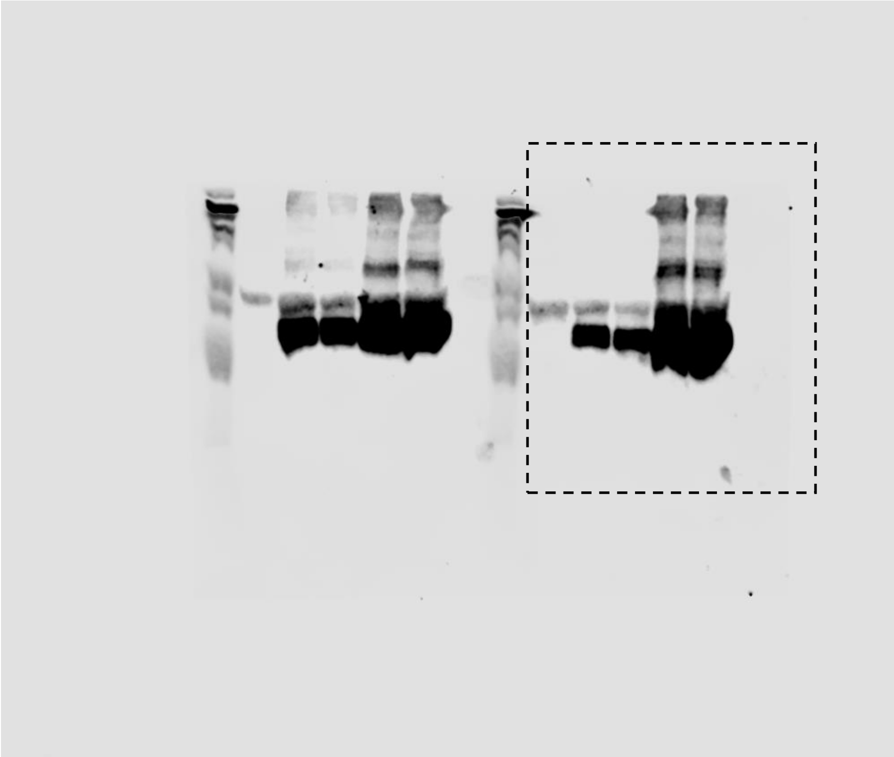

IP: FLAG
IB: FLAG

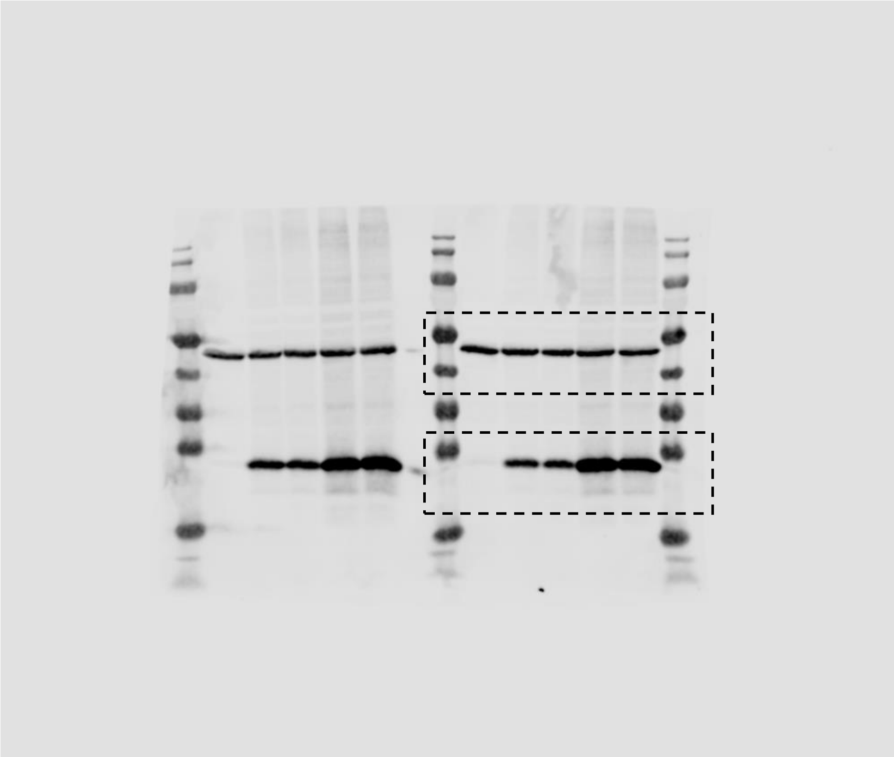

IB: γ-Tubulin, Load

IB: FLAG, Load

Figure 3A

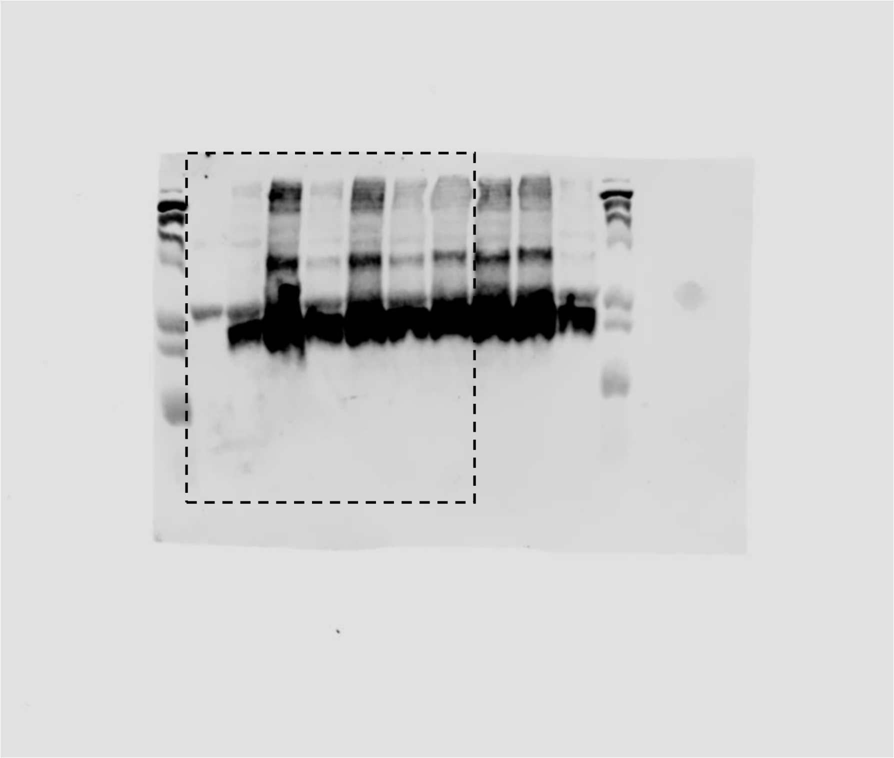

IP: FLAG
IB: FLAG

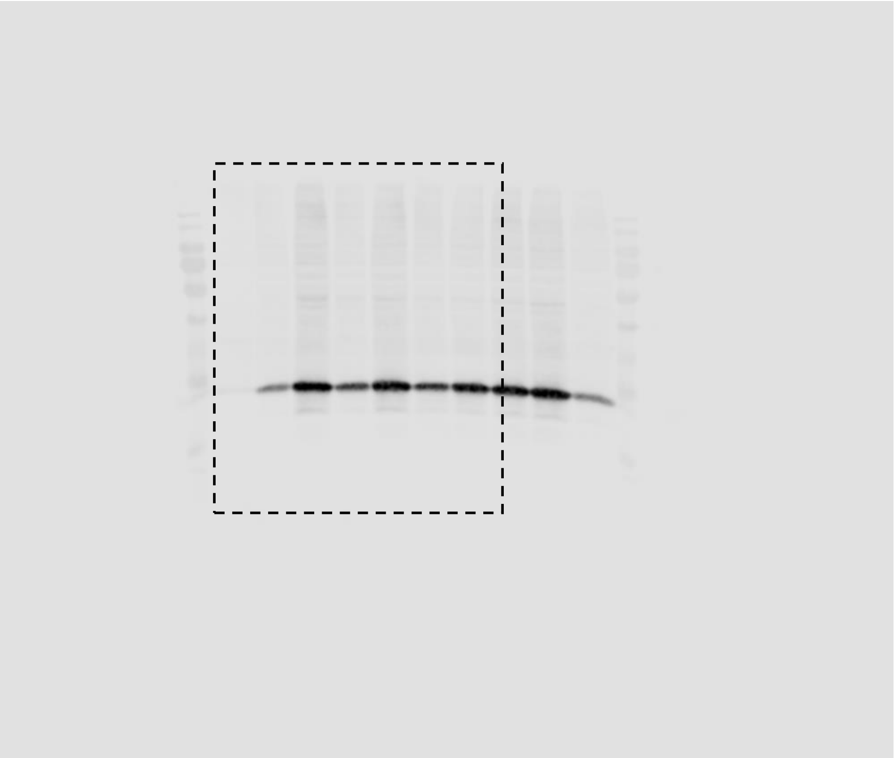

IB: FLAG,
Load

Figure 3A

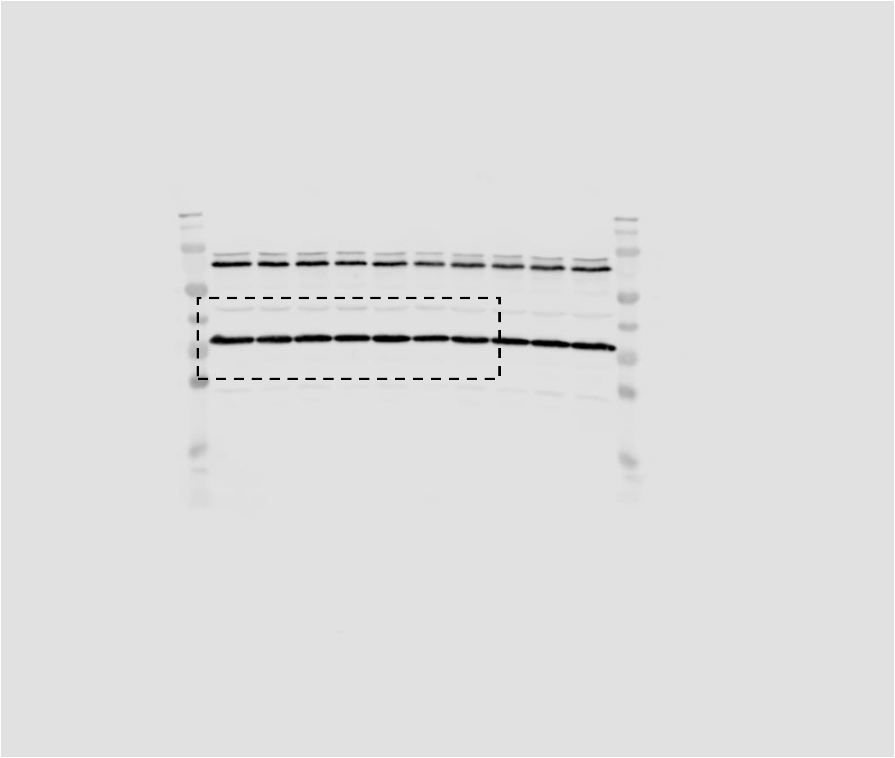

IB: GAPDH,
Load

Figure 3B

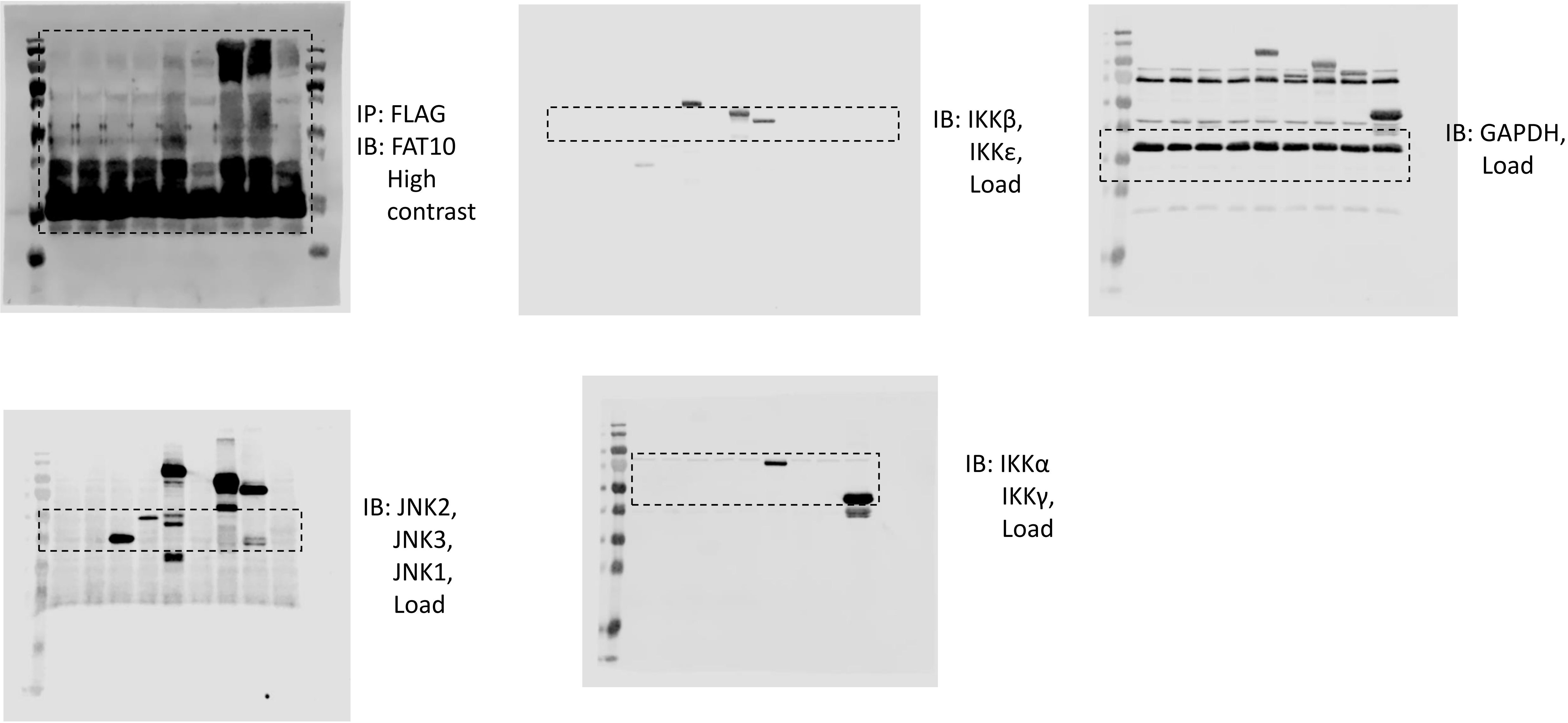
IP: FLAG
IB: FAT10
High contrast
IB: IKKβ,
IKKε,
Load
IB: GAPDH,
Load
IB: JNK2,
JNK3,
JNK1,
Load
IB: IKKα
IKKγ,
Load

Figure 3C

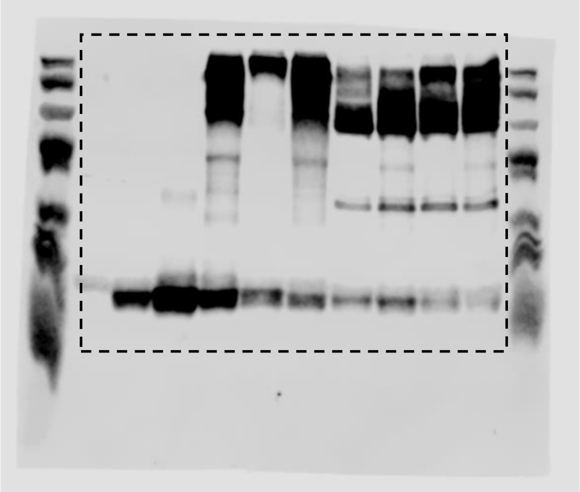

IP: FLAG
IB: FLAG

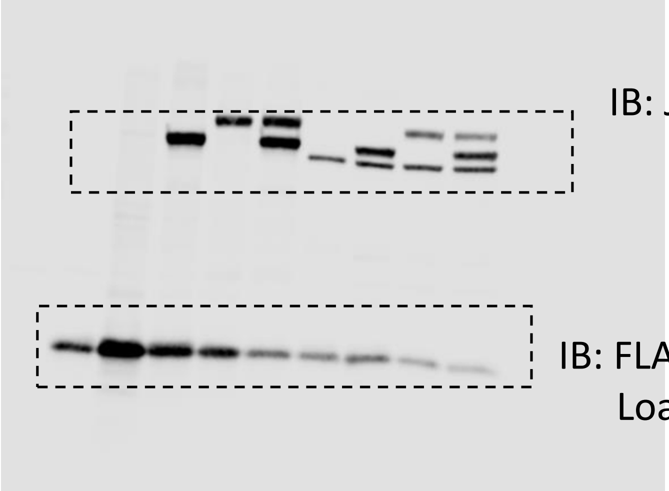

IB: JNK3,
IKKβ,
IKKε
Load

IB: FLAG,
Load

Figure 3D

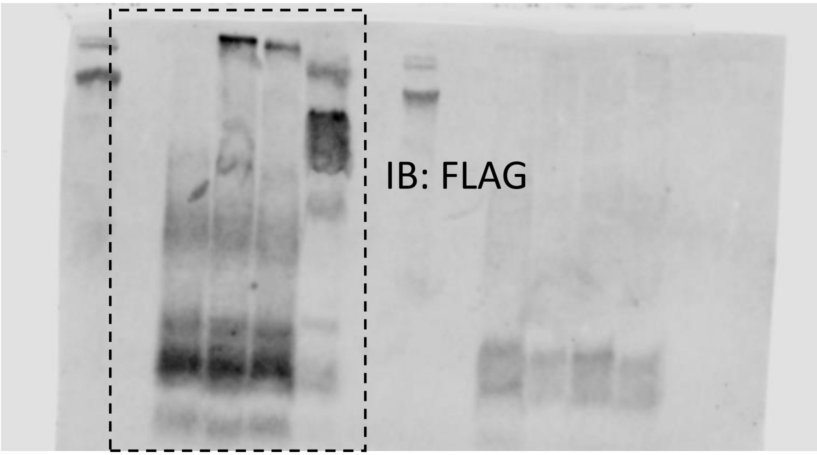
IB: FLAG

Figure 3E

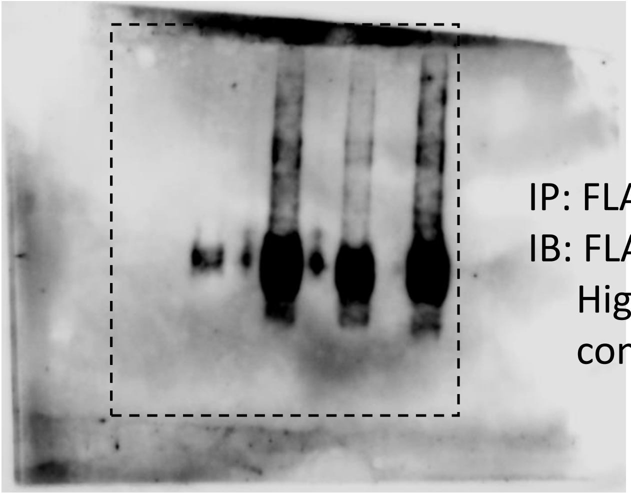
IP: FLAG
IB: FLAG
High contrast

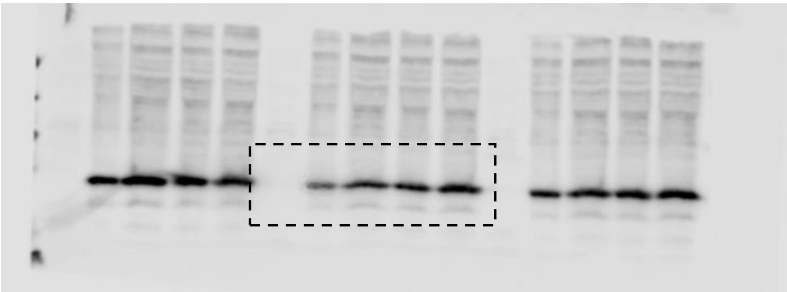
IB: FLAG,
Load

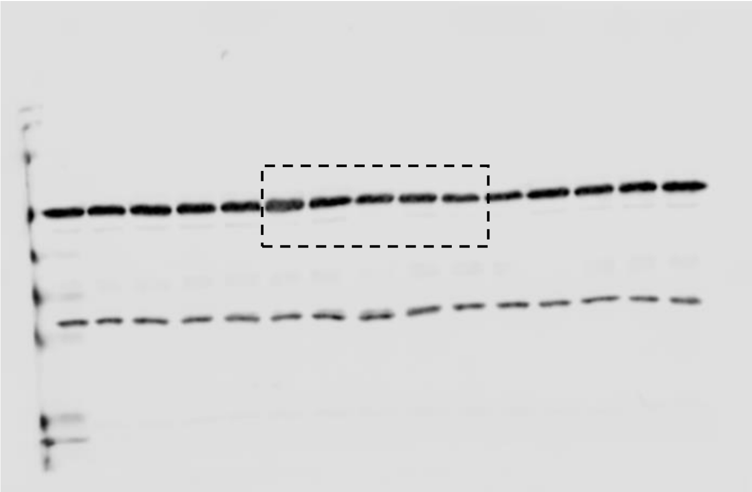
IB: α-tubulin,
Load

Figure 4B

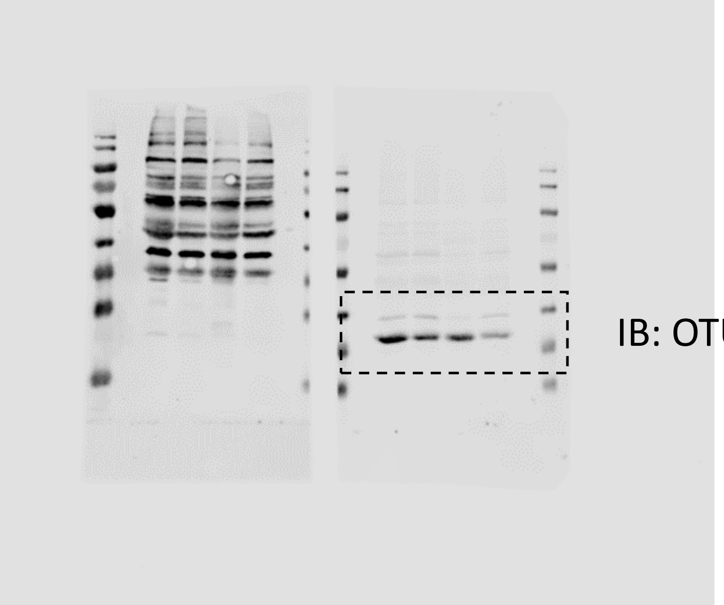

IB: OTUB1

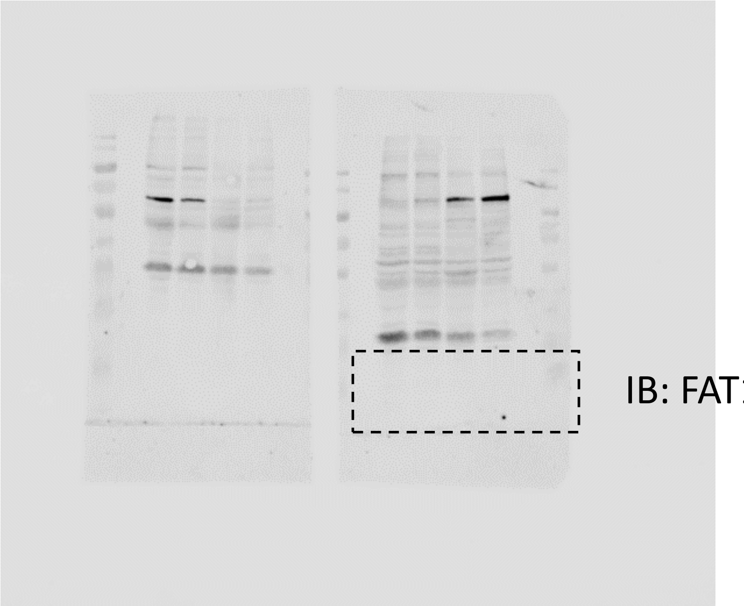

IB: FAT10

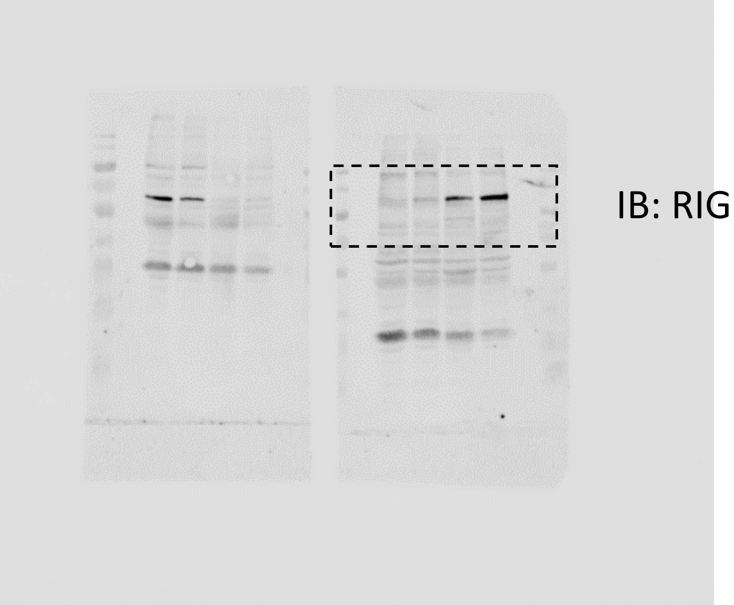

IB: RIGI

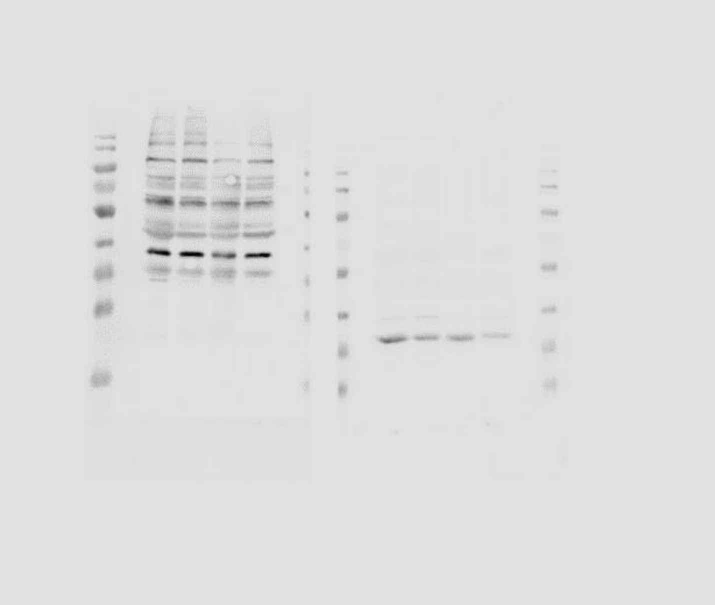

IB: α-tubulin

Figure 4C

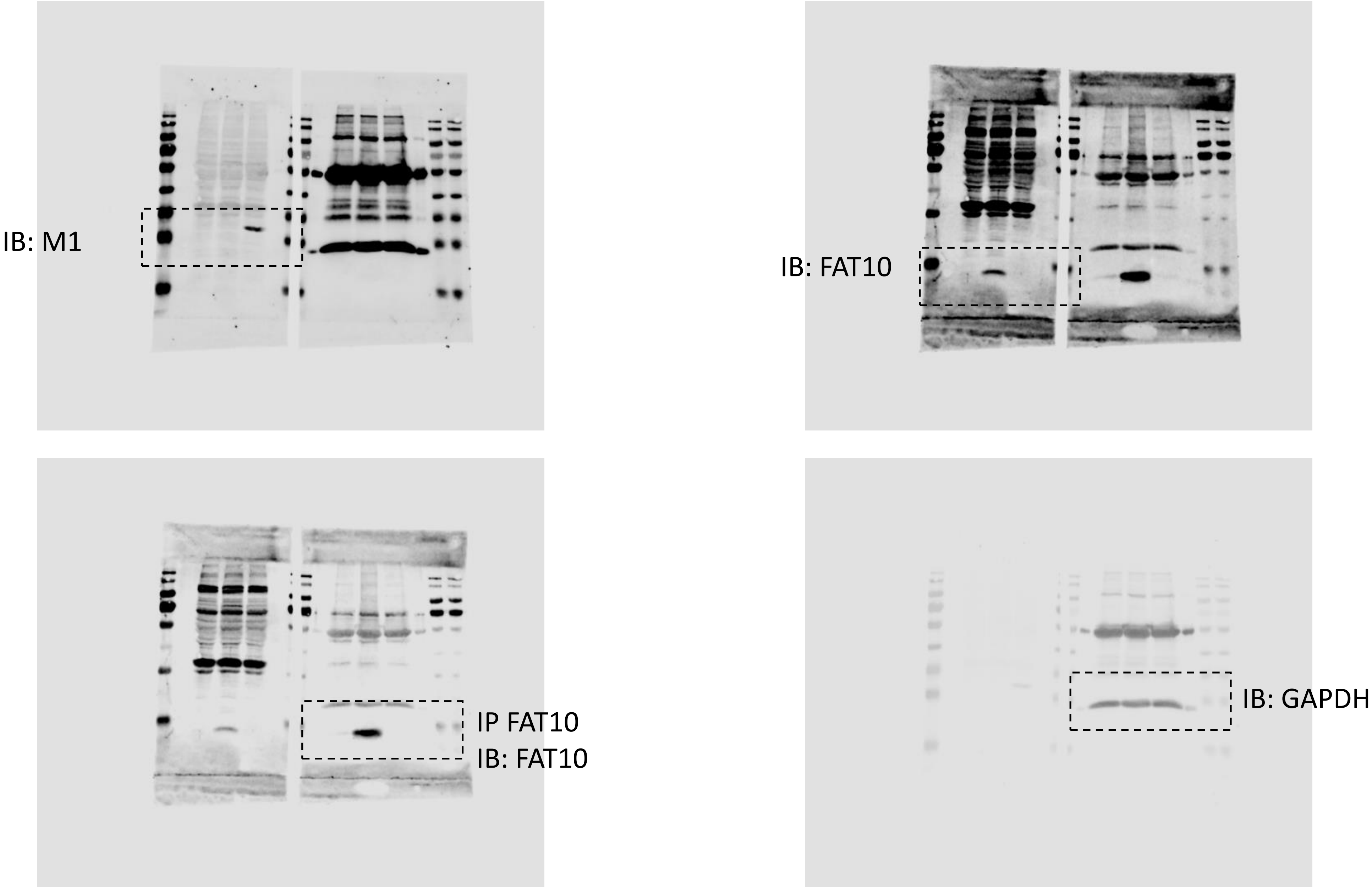

Figure 4D

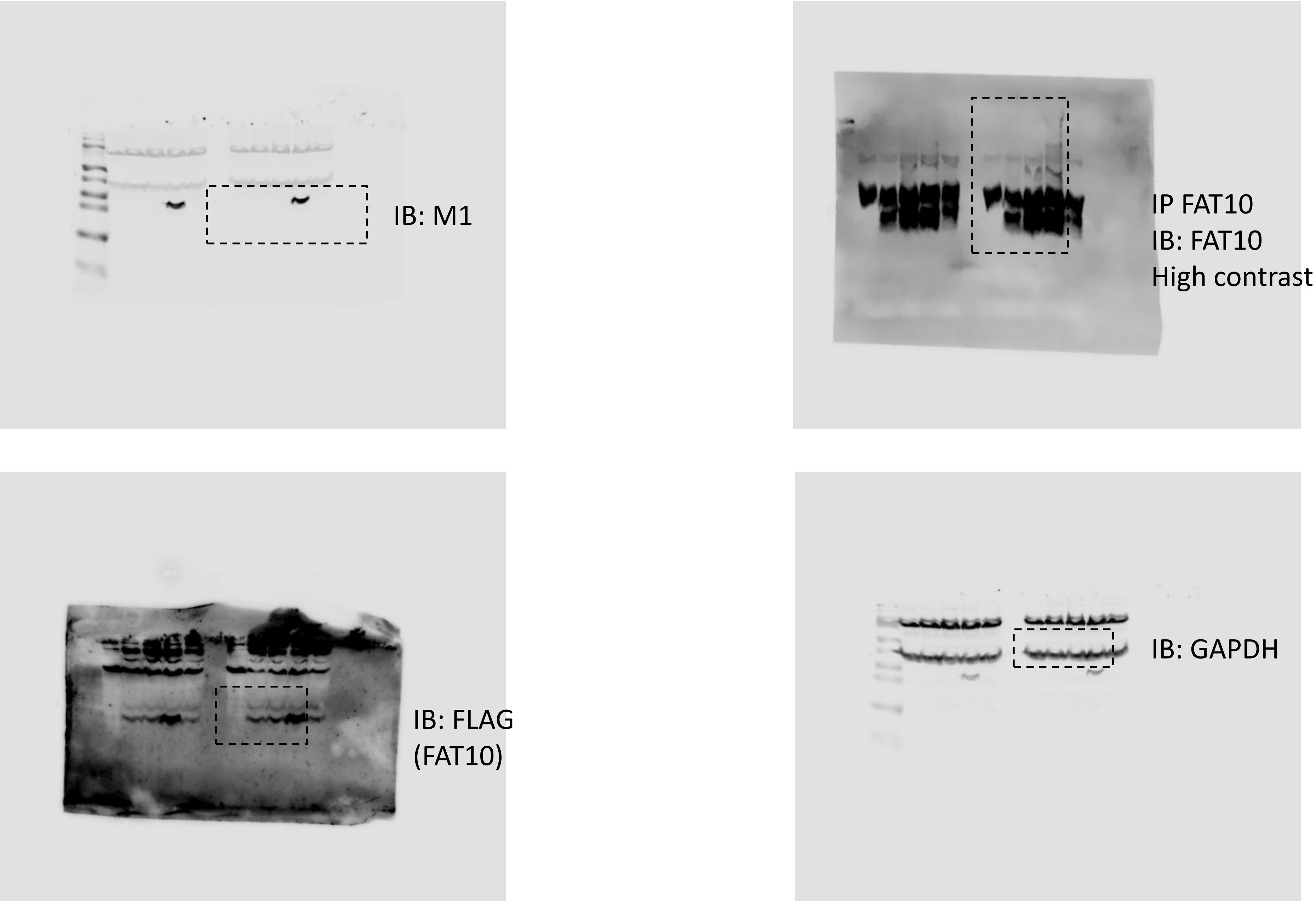

Figure 4E

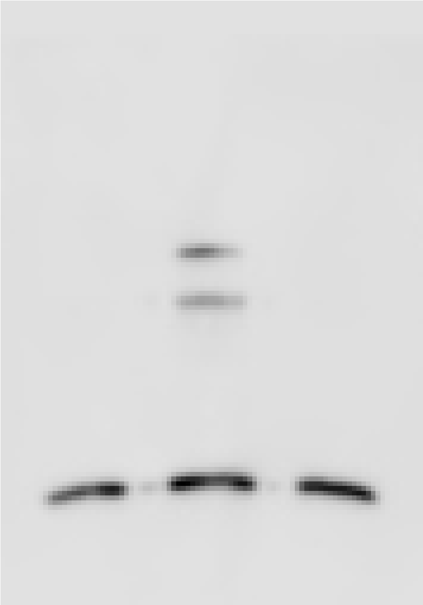

IB: FAT10

Figure 4F

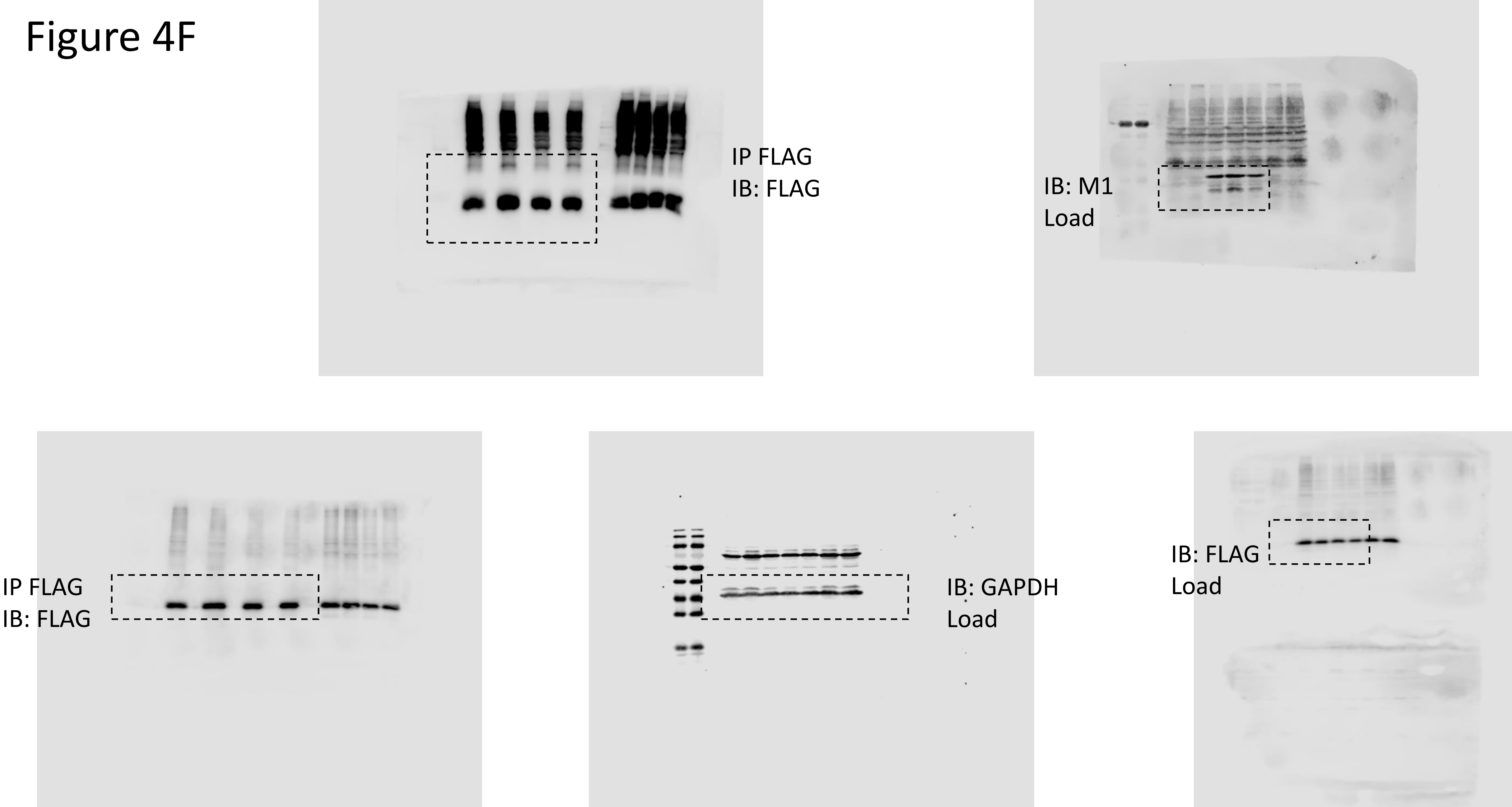
IP FLAG
IB: FLAG
IB: M1
Load
IP FLAG
IB: FLAG
IB: GAPDH
Load
IB: FLAG
Load

Figure 5A

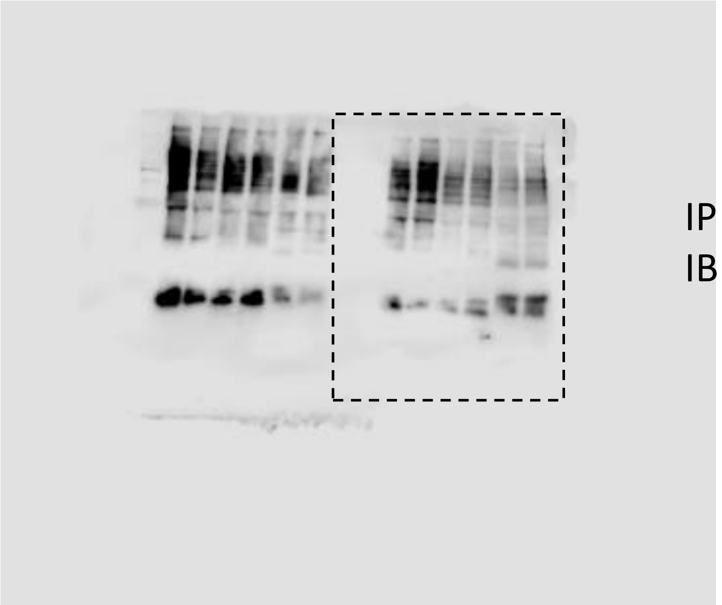

IP FLAG
IB: FLAG

IB: FLAG
Load

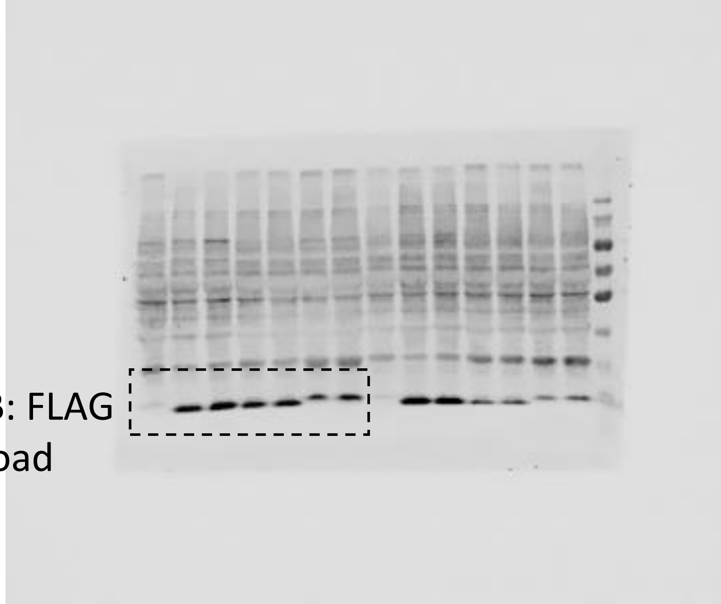

IB: GAPDH
Load

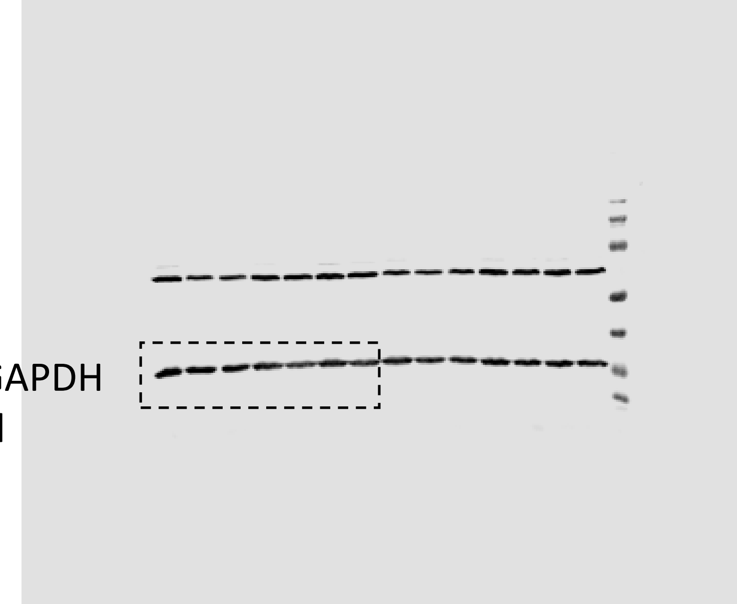

Figure 5C

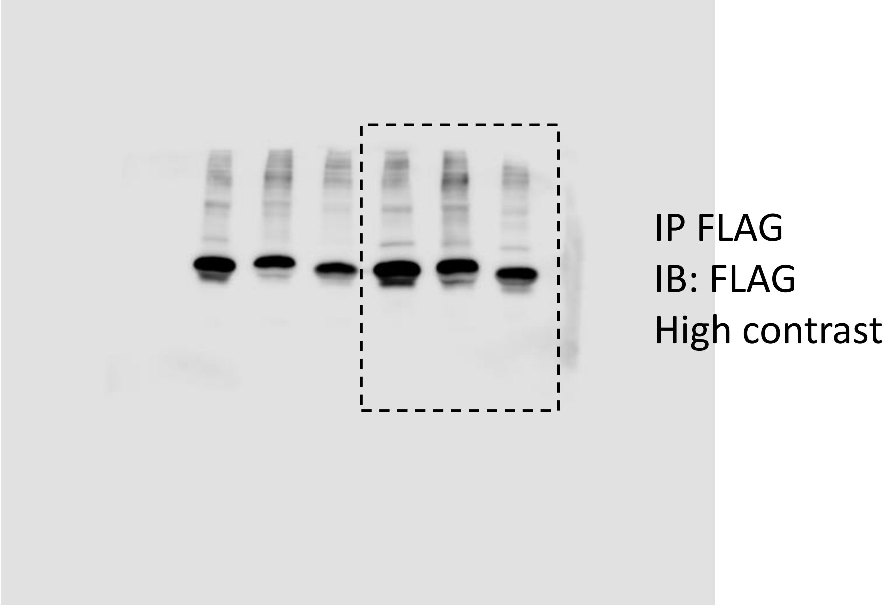

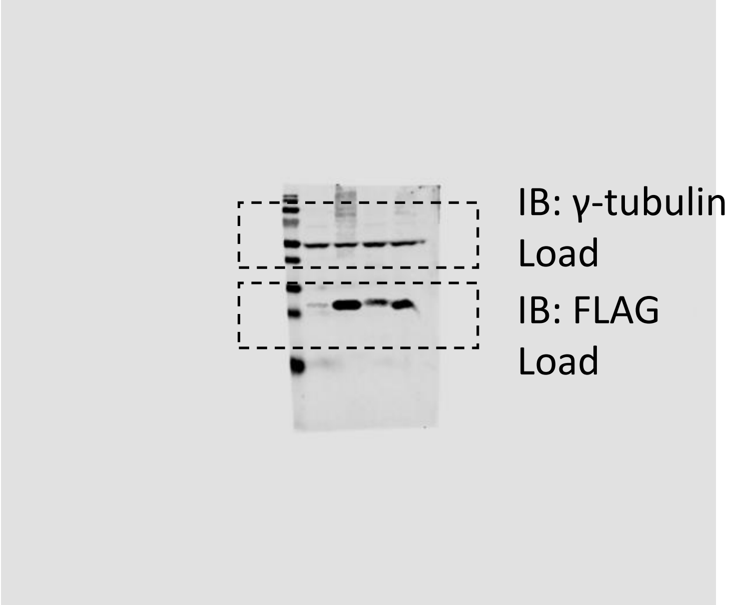

Figure 5D

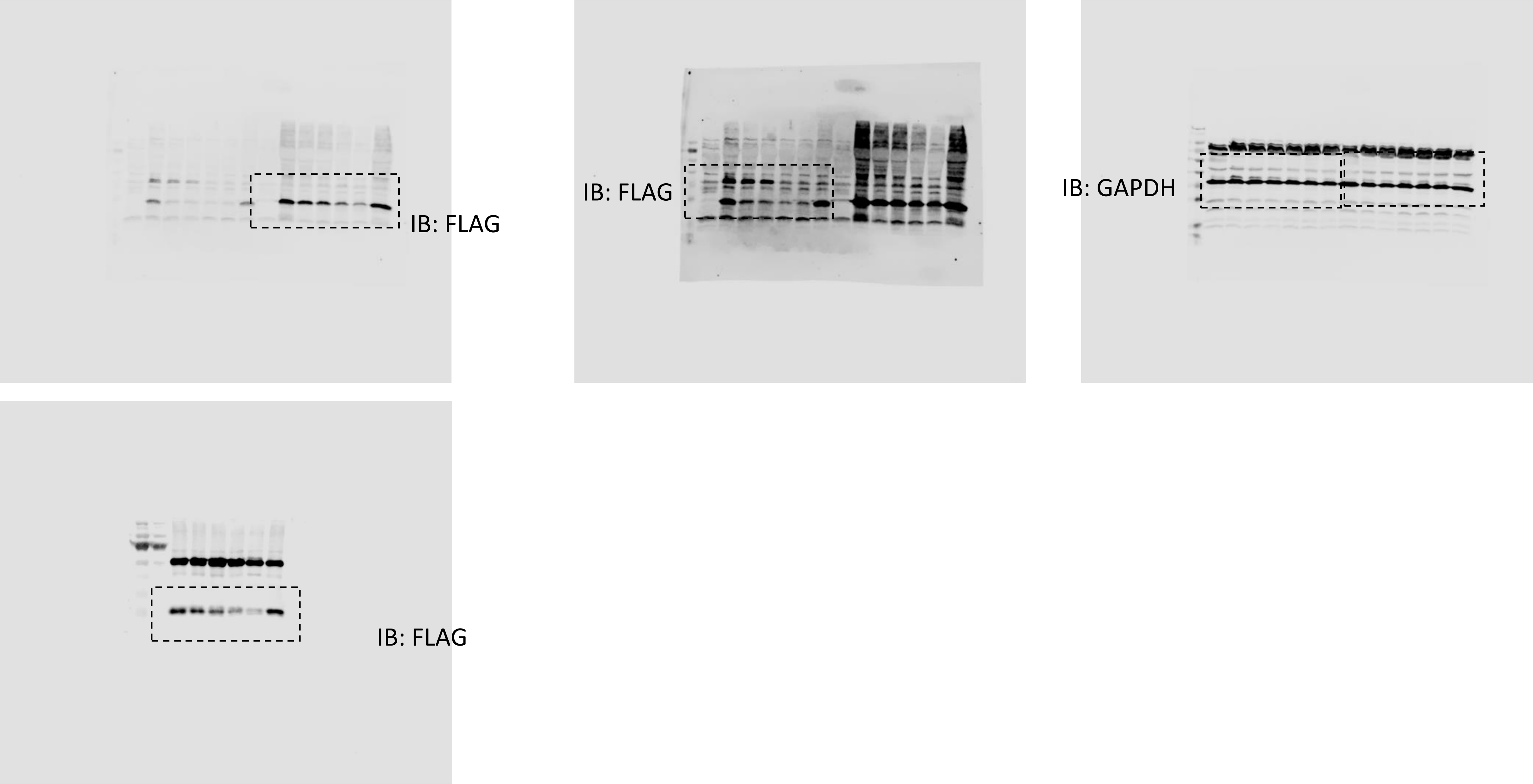

Figure 6D

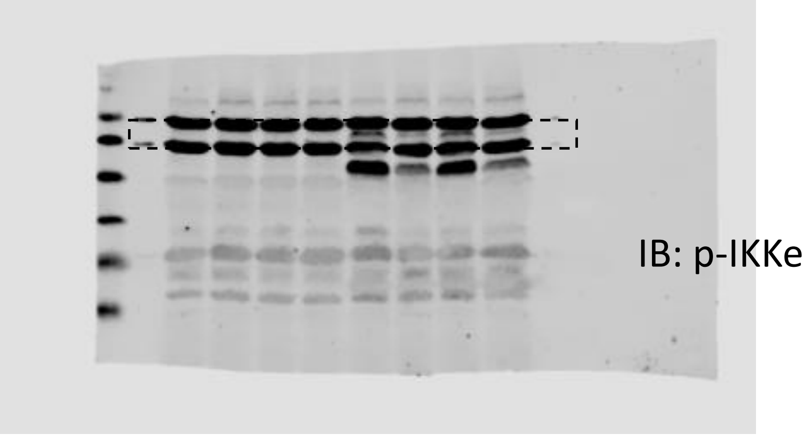

IB: p-IKKe

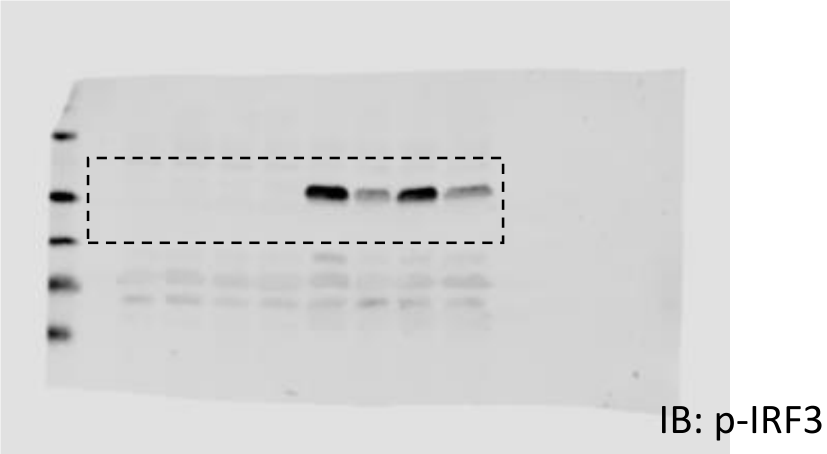

IB: p-IRF3

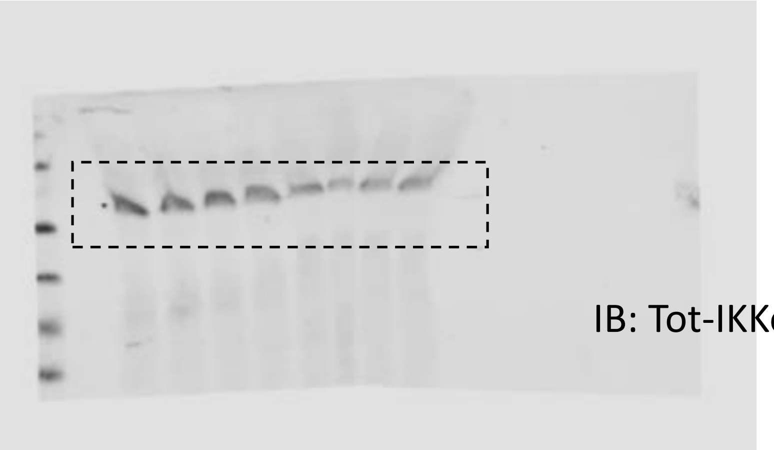

IB: Tot-IKKe

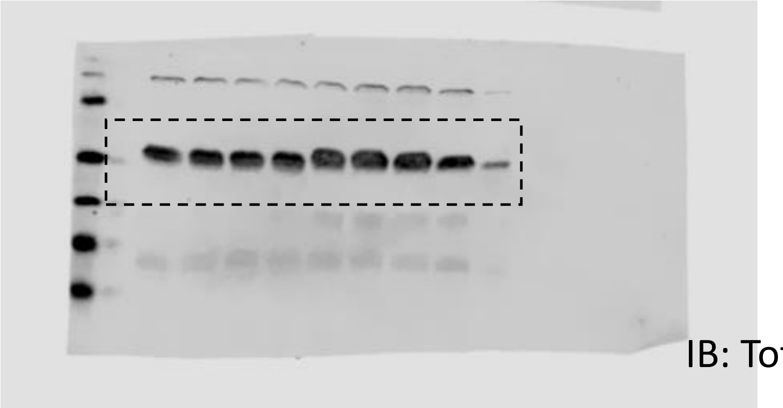

IB: Tot-IRF3

Figure 6D

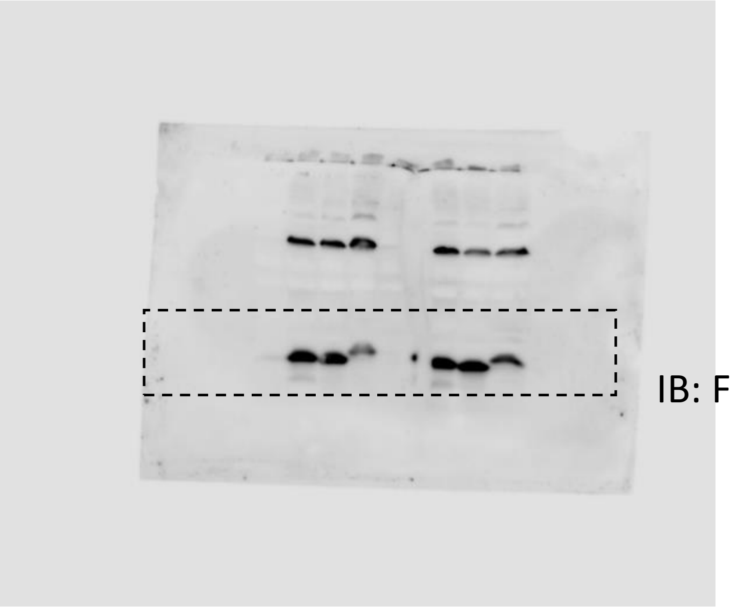

IB: FLAG-FAT10

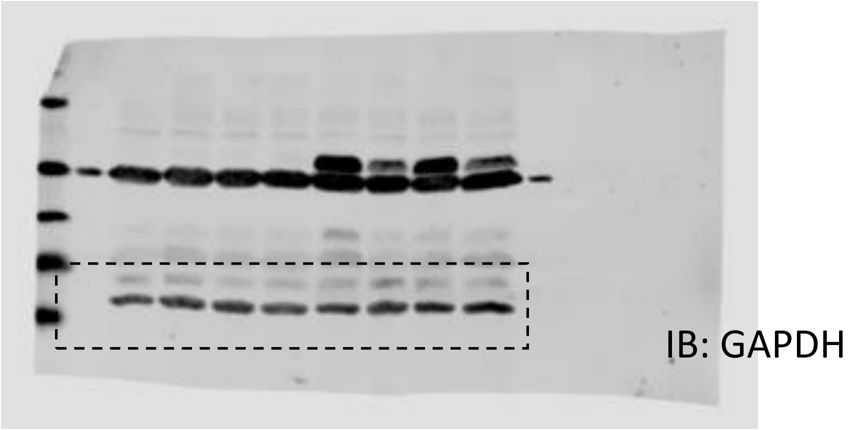

IB: GAPDH

Figure 7A

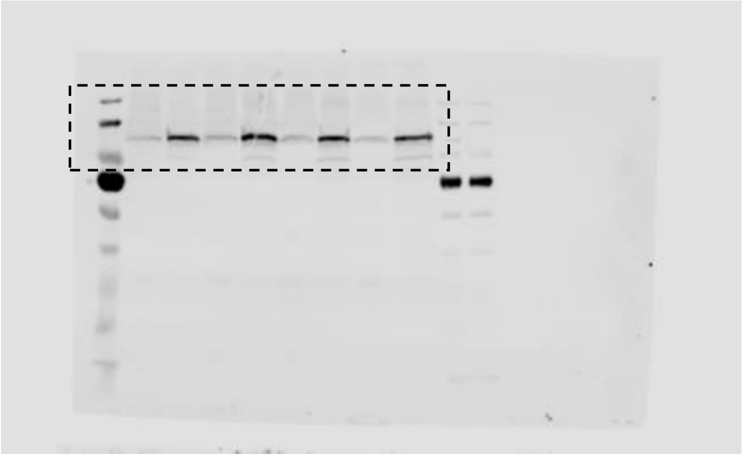

IB: RIG-I

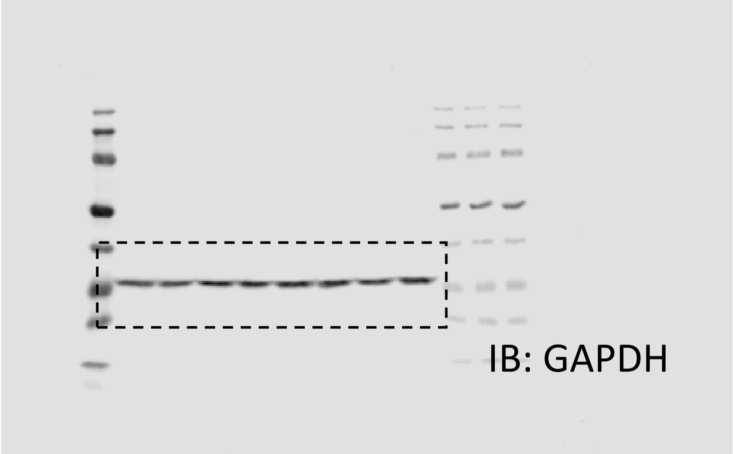

IB: GAPDH

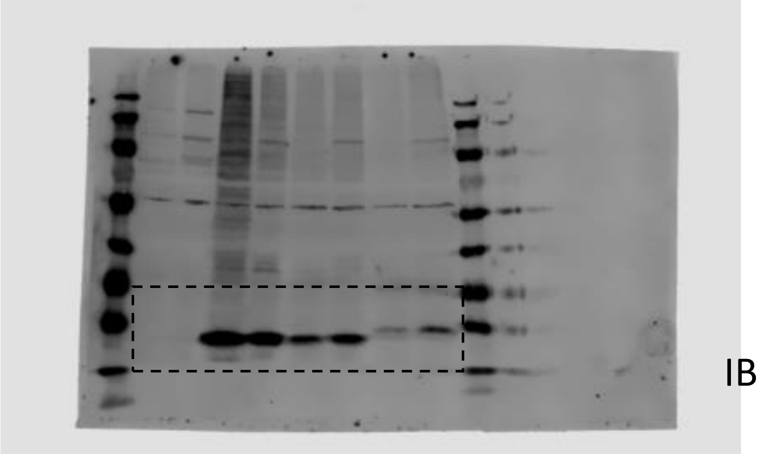

IB: FLAG-FAT10

Figure 7C

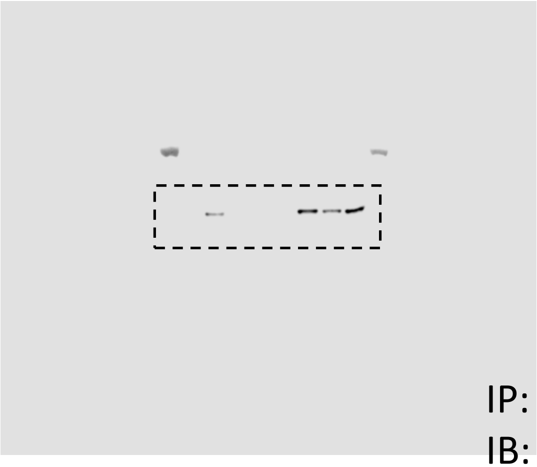

IP: FLAG
IB: HA

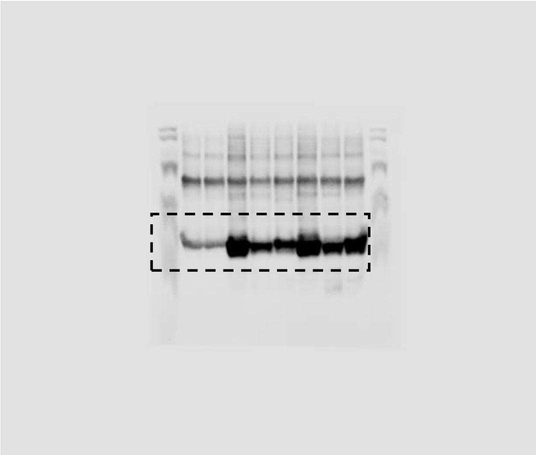

IP: FLAG
IB: FLAG

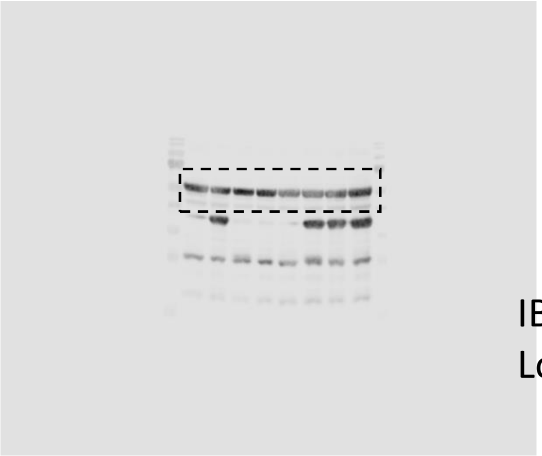

IB: γ-tubulin
Load

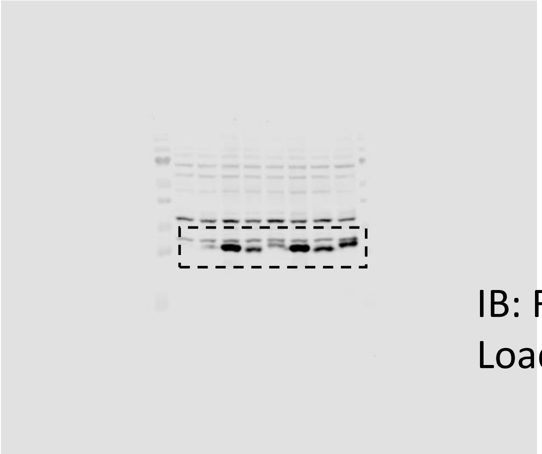

IB: FLAG-FAT10
Load

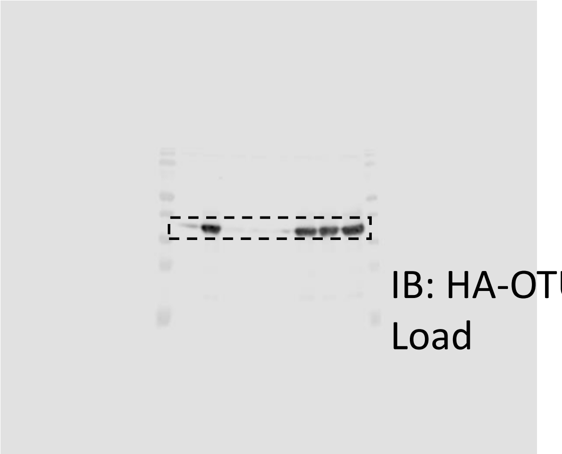

IB: HA-OTUB1
Load

Figure 7D

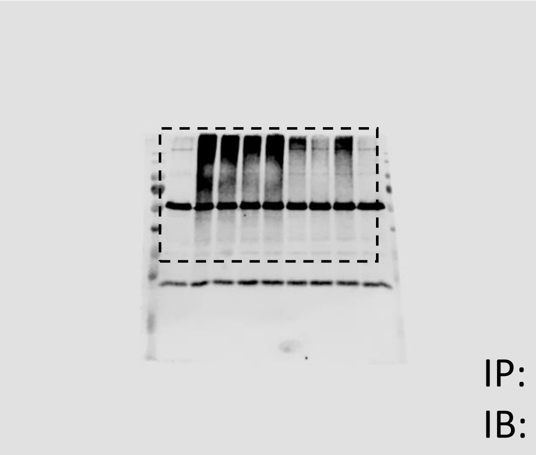

IP: TRAF3
IB: HA

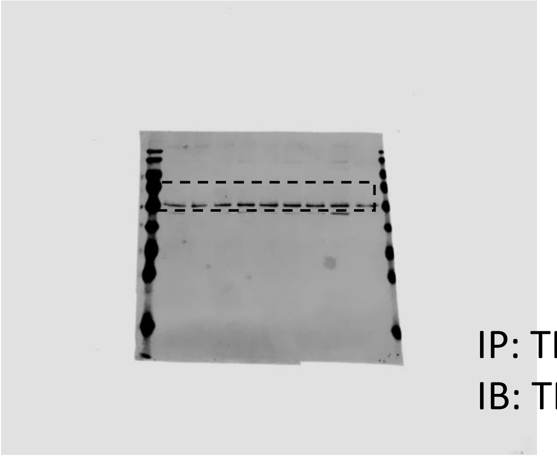

IP: TRAF3
IB: TRAF3

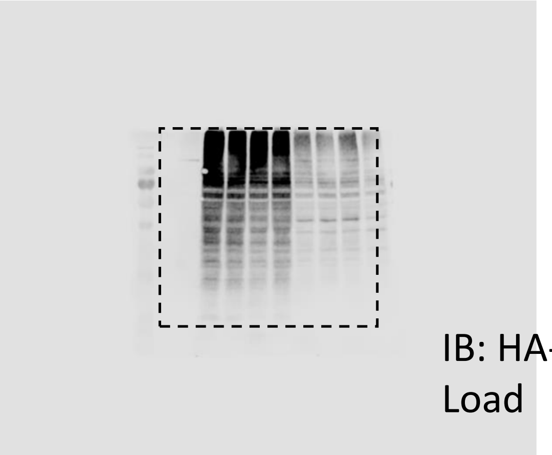

IB: HA-OTUB
Load

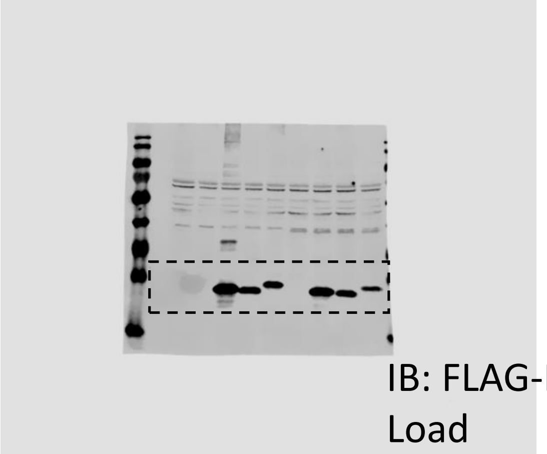

IB: FLAG-FAT10
Load

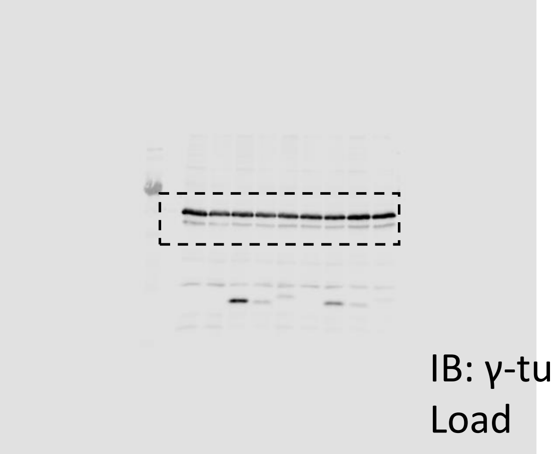

IB: γ-tubulin
Load

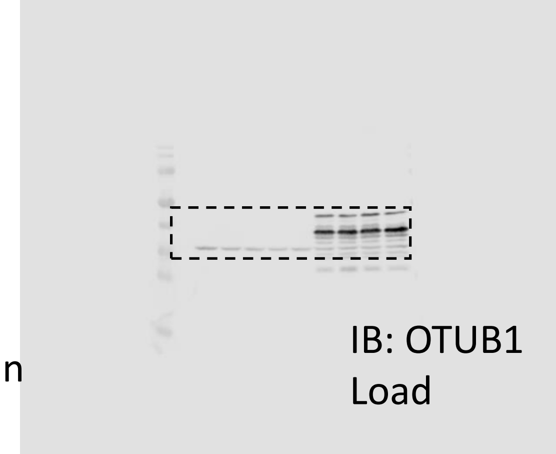

IB: OTUB1
Load

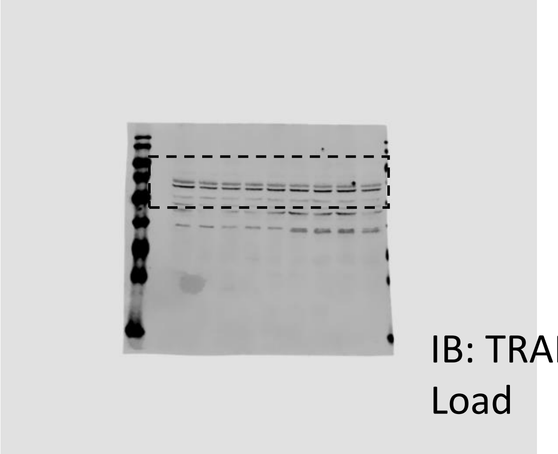

IB: TRAF3
Load

Figure S3

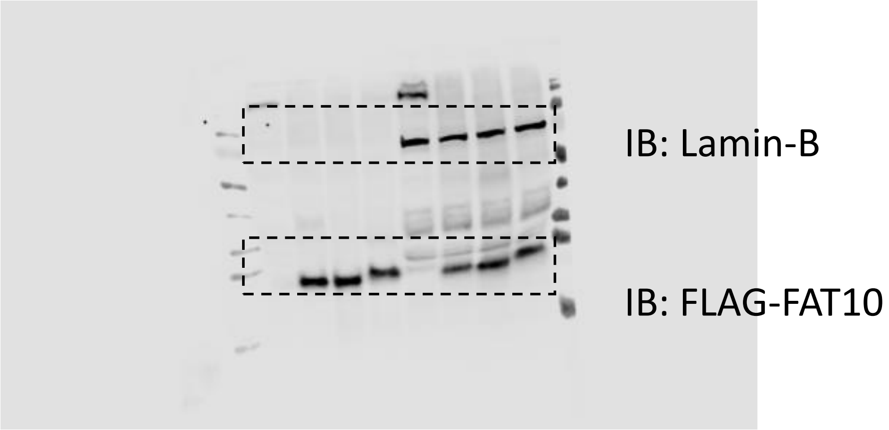

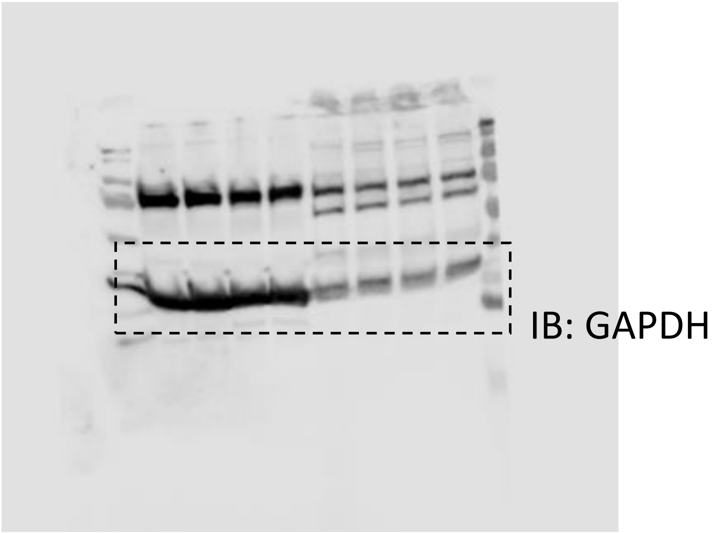

Figure S4A

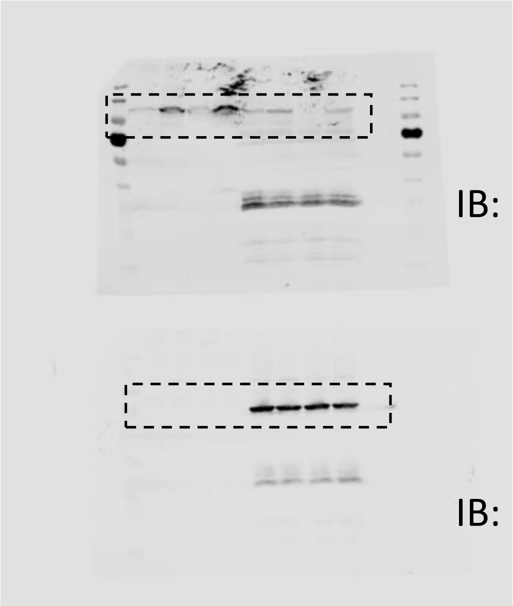

IB: RIG-I

IB: Lamin-B

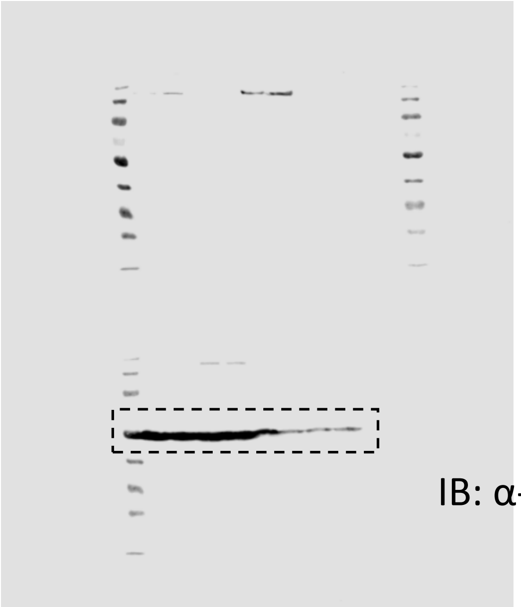

IB: α-tubulin

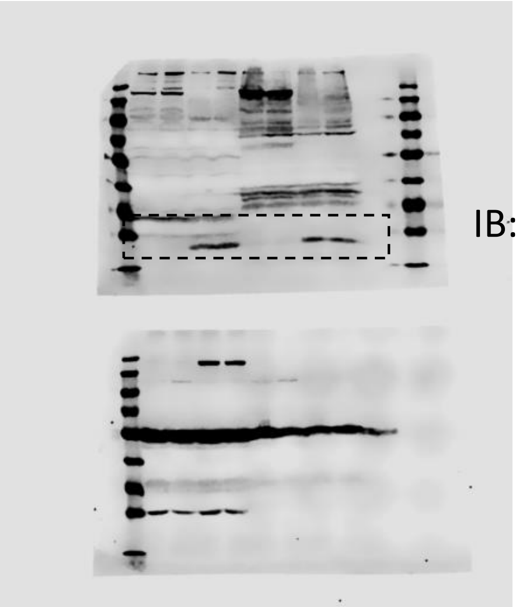

IB: FLAG-FAT10

Figure S4B

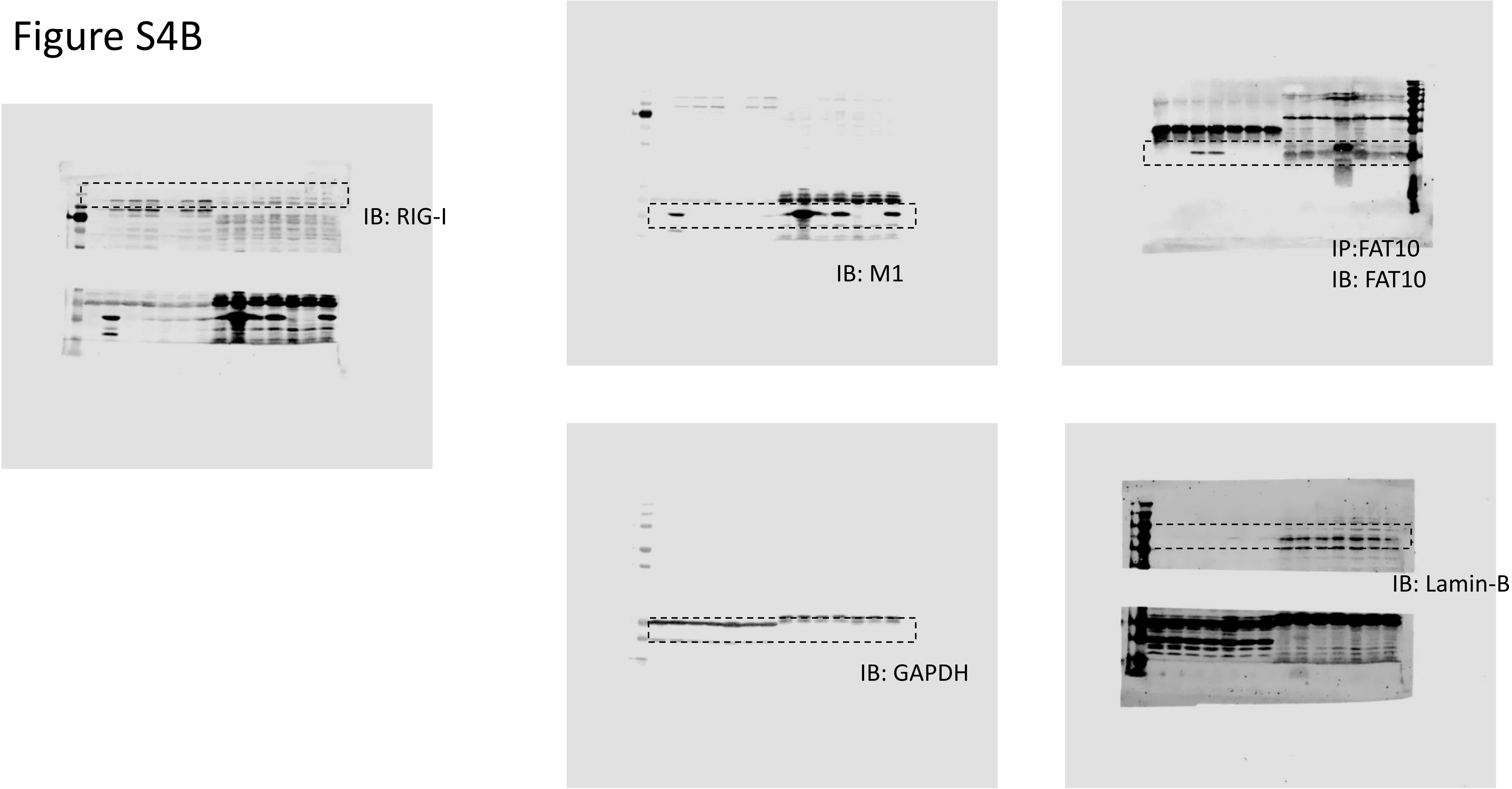

Figure S5A

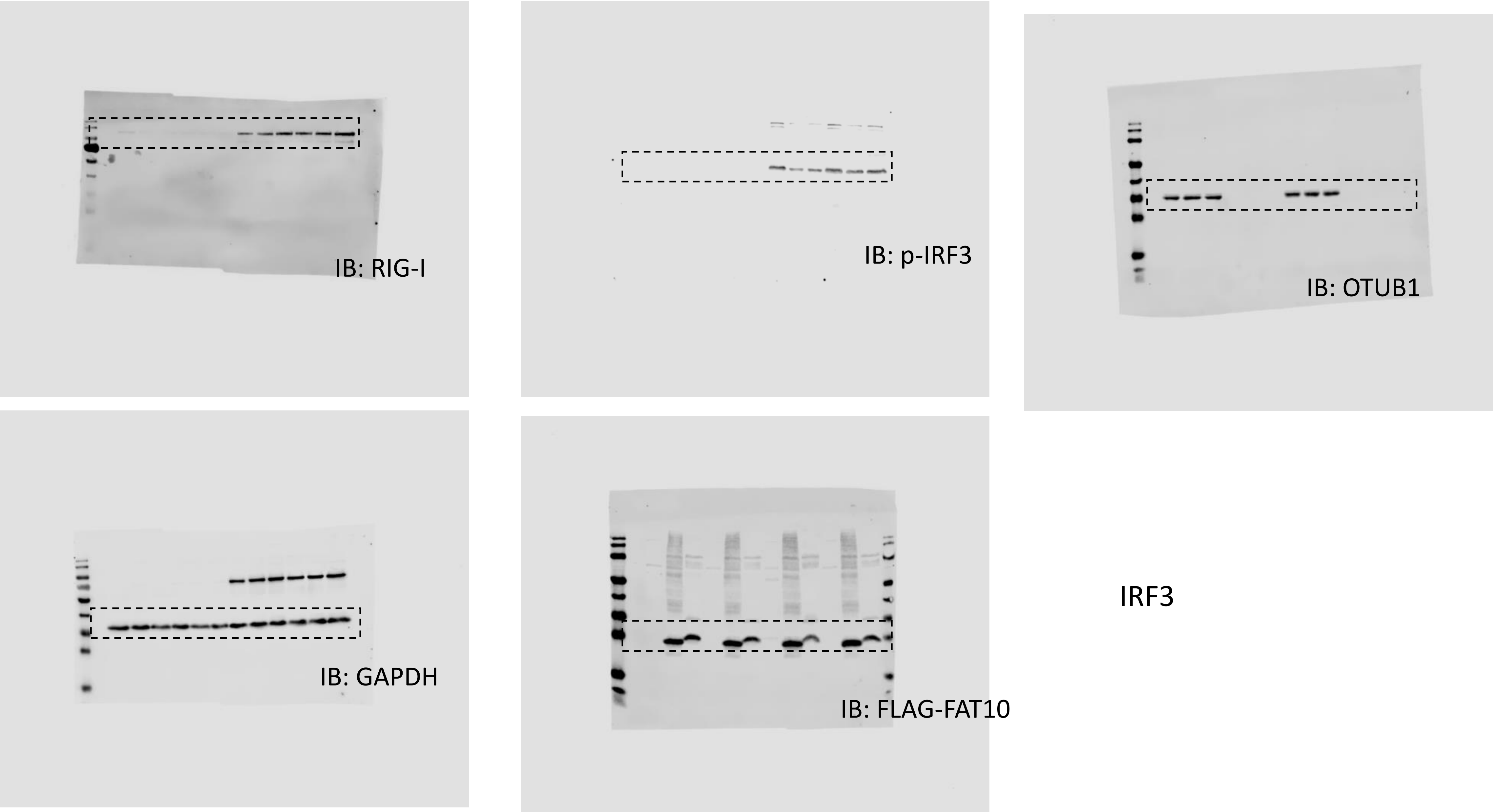

Figure S5B

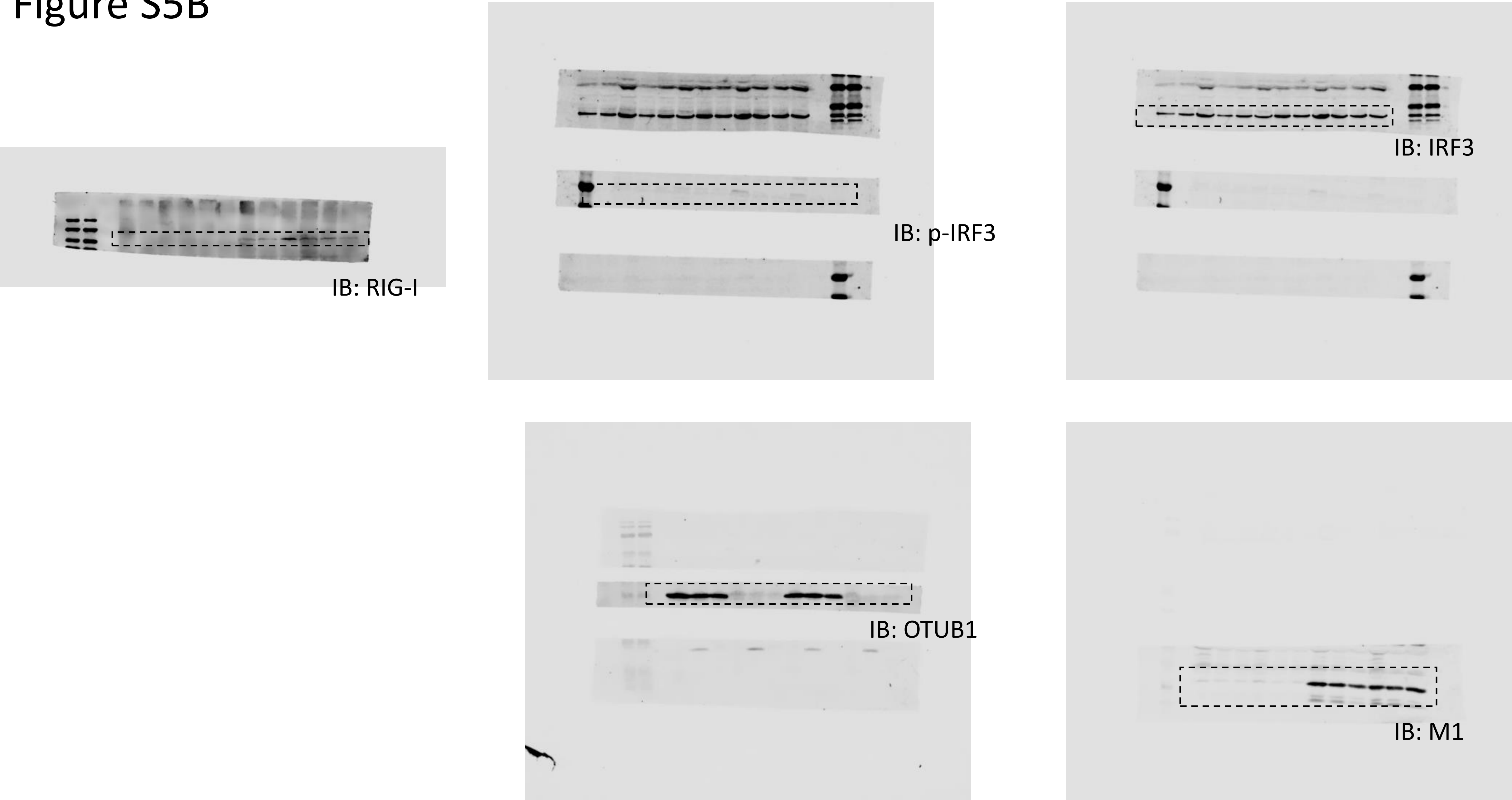

Figure S5B

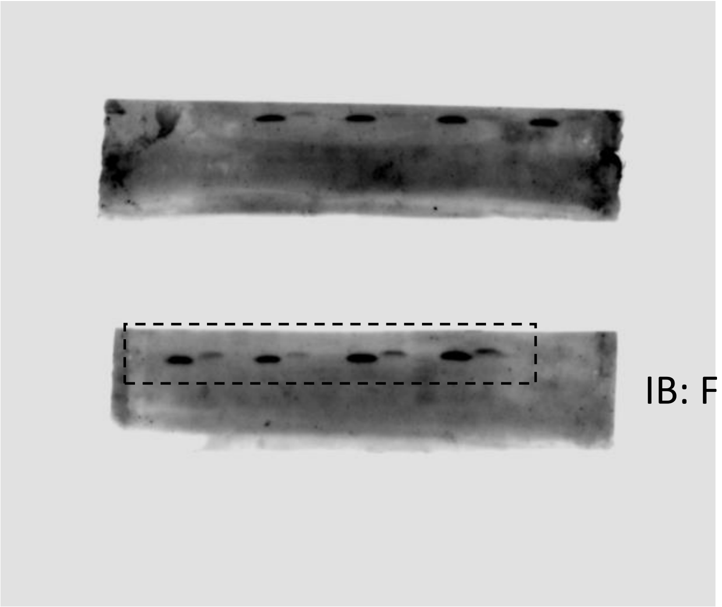

IB: FLAG-FAT10

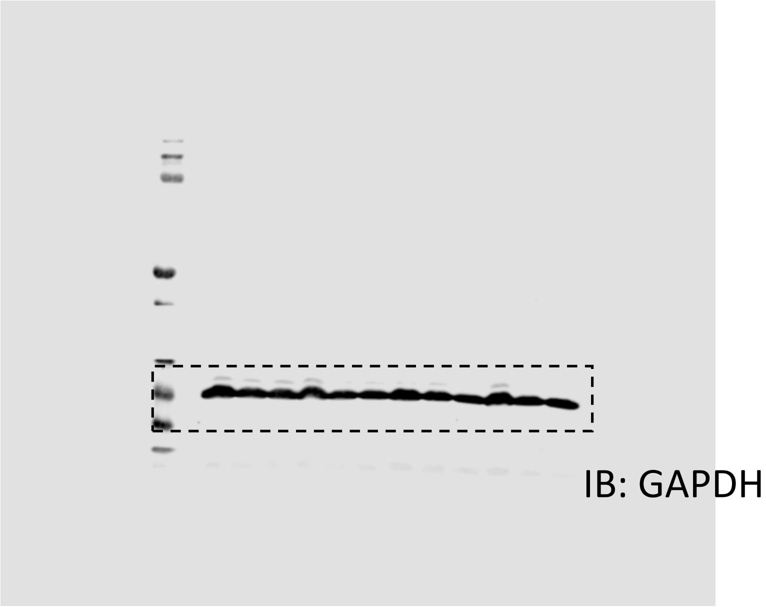

IB: GAPDH

Figure S5B

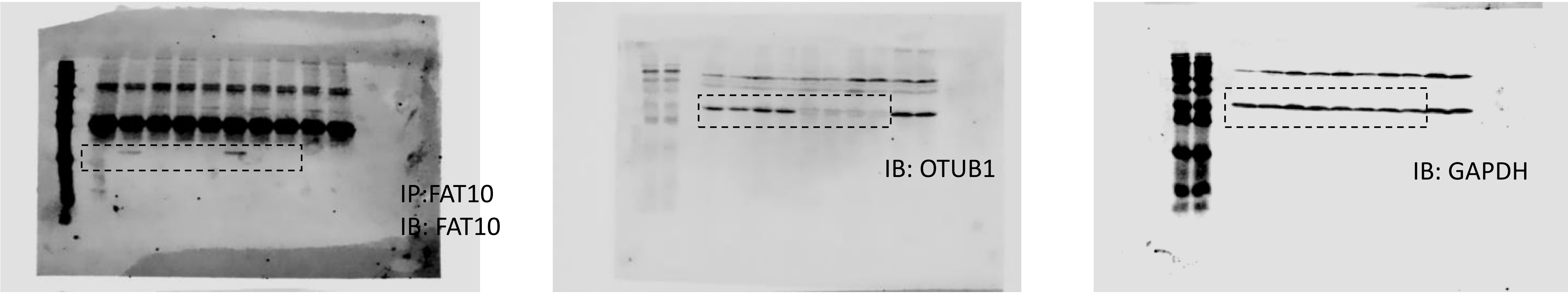

Supplement: Supplementary file 1 [file LSA-2021-01282_SdataF1_F2_F3_F4_F5_F6_F7_FS3_FS4_FS5.pdf]
